# Longitudinal study of the relationship between patients’ medication adherence and quality of life outcomes and illness perceptions and beliefs about cardiac rehabilitation

**DOI:** 10.1186/s12872-020-01378-4

**Published:** 2020-02-11

**Authors:** Patricia Thomson, Gordon F. Rushworth, Federico Andreis, Neil J. Angus, Andrea R. Mohan, Stephen J. Leslie

**Affiliations:** 1 Faculty of Health Sciences and Sport, University of Stirling, Stirling, FK94LA Scotland, UK; 2 Highland Pharmacy Education & Research Centre, Centre for Health Science, Old Perth Road, Inverness, IV2 3JH Scotland, UK; 3 School of Health, Social Care and Life Sciences, University of the Highlands and Islands, Centre for Health Science, Old Perth Road, Inverness, IV2 3JH Scotland, UK; 4 School of Nursing and Health Sciences, University of Dundee, Dundee, Scotland, UK; 5 Cardiac Unit, Raigmore Hospital, NHS Highland, Old Perth Road, Inverness, IV2 3UJ Scotland, UK

**Keywords:** Cardiac rehabilitation, Beliefs, Illness perceptions, Medication adherence, Quality of life

## Abstract

**Background:**

Adherence to medication regimens is essential for preventing and reducing adverse outcomes among patients with coronary artery disease (CAD). Greater understanding of the relation between negative illness perceptions, beliefs about cardiac rehabilitation (CR) and medication adherence may help inform future approaches to improving medication adherence and quality of life (QoL) outcomes. The aims of the study are: 1) to compare changes in illness perceptions, beliefs about CR, medication adherence and QoL on entry to a CR programme and 6 months later; 2) to examine associations between patients’ illness perceptions and beliefs about CR at baseline and medication adherence and QoL at 6 months.

**Methods:**

A longitudinal study of 40 patients with CAD recruited from one CR service in Scotland. Patients completed the Medication Adherence Report Scale, Brief Illness Perception Questionnaire, Beliefs about CR questionnaire and the Short-Form 12 Health Survey. Data were analysed using the Wilcoxon Signed Ranks test, Pearson Product Moment correlation and Bayesian multiple logistic regression.

**Results:**

Most patients were men (70%), aged 62.3 mean (SD 7.84) years. Small improvements in ‘perceived suitability’ of CR at baseline increased the odds of being fully adherent to medication by approximately 60% at 6 months. Being fully adherent at baseline increased the odds of staying so at 6 months by 13.5 times. ‘Perceived necessity, concerns for exercise and practical barriers’ were negatively associated with reductions in the probability of full medication adherence of 50, 10, and 50%. Small increases in concerns about exercise decreased the odds of better physical health at 6 months by about 50%; and increases in practical barriers decreased the odds of better physical health by about 60%. Patients perceived fewer consequences of their cardiac disease at 6 months.

**Conclusions:**

Patients’ beliefs on entry to a CR programme are especially important to medication adherence at 6 months. Negative beliefs about CR should be identified early in CR to counteract any negative effects on QoL. Interventions to improve medication adherence and QoL outcomes should focus on improving patients’ negative beliefs about CR and increasing understanding of the role of medication adherence in preventing a future cardiac event.

## Background

Patients with a new diagnosis of a cardiac condition are highly likely to have a change in medication for what is most likely a long-term condition. Medication adherence is vital for patients to obtain the best mortality and morbidity benefits from these medication changes [1]. Adherence is more than simply a count of medications compliance; the term adherence encapsulates the concept that there may be reasons why a patient may be unable or may be unwilling to take a medication [2–4]. It is important that prescribers are aware of factors which influence adherence to medication so that these might be explored during the consultation, so that a management plan can be agreed between the prescriber and the patient to attain concordance – the point at which a patient and prescriber are working to the same identified outcomes [5].

Adherence for long term conditions is poor; the World Health Organization predicted compliance with medication for long term conditions at around 50% [6]. There is a major public health concern here, both in terms of populations of patients who are, through lack of adherence, on suboptimal treatment regimen; as well as to healthcare providers such as the NHS within the UK who are paying for medications which are going to waste. A recent estimate from NHS England puts the total figure for pharmaceutical waste at £300 million per annum [4]. Symptoms which lead to the diagnosis of heart disease may differ in the extreme – silent myocardial infarction (MI), compared to ST elevation myocardial infarction (STEMI), compared to stable angina, for example. What is not known is what effect illness perceptions and treatment beliefs, i.e. beliefs about CR have on patient’s adherence, as well as what effect their experiences of CR have on shaping this. Furthermore, there is a need to understand the effect that time has on patient’s beliefs and QoL, and whether these effects wane or are maintained.

## Method

The aims of the study are: 1) to compare changes in illness perceptions, beliefs about CR, medication adherence and QoL on entry to a CR programme and 6 months later; 2) to examine associations between patients’ illness perceptions and beliefs about CR at baseline and medication adherence and QoL at 6 months.

### Study design

This was a longitudinal study of patients with coronary artery disease.

### Setting and participants

Data were collected using a convenience sample of patients attending a hospital-based CR service in one NHS Board in northern Scotland between 2014 and 2015. The number of study subjects was determined by practical considerations; further details on the sample size and response rates are given in the results section. Eligible patients were aged 45 years or over, had a confirmed medical diagnosis of CAD, and were on stable doses of cardiac prevention medication. Patients were excluded if there were any major co-morbidities such as stroke or cancer, or psychological or communication limitations likely to affect their ability to give informed consent.

### Recruitment and data collection

Patients were recruited on their initial attendance at the hospital based CR programme. Study information and consent forms were distributed by the CR specialists, in accordance with the inclusion and exclusion criteria. After receipt of the signed consent forms, the researcher (PT) posted questionnaire packs to the participant’s home address or provided a link to the Bristol on-line survey for completion depending on individual preference. Completed questionnaires were returned to the researcher (PT) by post or email. A reminder letter was sent after 2 weeks. After 6 months the participants were contacted again to complete follow-up questionnaires.

### Ethical approval

This study was approved by the University of Stirling Ethics and Research Committee and the National Research and Ethics Committee (NRES), North of Scotland (Rec ref. 13/NS/0152 (IRAS project ID: 133236). A written consent was obtained from all participants in the study.

### Instruments

#### Illness perceptions

Patients’ illness perceptions were assessed using the Brief Illness Perception Questionnaire (B-IPQ) [7]. The B-IPQ consists of eight separate items, i.e. consequences, timeline, personal control, treatment control, identity, illness concern, coherence (understanding) and emotional representation relating to the patient’s illness (i.e. CAD), each scored from 0 to 10. The cumulative score for items 1–8 gives a score range of 0 to 80. In order to compute the overall score, items 3, 4, and 7 were reverse coded. A higher total score reflects a more threatening (negative) view of the illness [7]. The B-IPQ also has a causal representation item (item 9) which requires an open-ended response (not reported in this paper). The B-IPQ has demonstrated good validity and test-retest reliability in research [7–10]. In the study, Cronbach’s alpha for the B-IPQ total score was 0.75 for patients.

#### Beliefs about cardiac rehabilitation

Patients’ beliefs about CR were assessed using the Beliefs about Cardiac Rehabilitation questionnaire (BCR-Q) [11], a 13 item self-administered tool consisting of four sub-scales: perceived necessity, 5 items; concerns about exercise, 3 items; practical barriers, 3 items; and perceived suitability, 2 items. All items on the BCR-Q are rated on a 5-point Likert scale from 1 = strongly disagree to 5 = strongly agree, with the exception of one item on the necessity scale i.e. ‘some aspects of the CR programme are unnecessary for me’, which is reversed scored. For each sub-scale, the scores are summed and means obtained: necessity (range 9–21); concerns about exercise (range 3–15); practical barriers (range 3–15); and perceived suitability (range 2–10). The BCR-Q has been shown to be a valid and reliable measure of beliefs about CR [11, 12]. Cronbach’s alpha for the 4 sub-scales of BCR-Q range from 0.68–0.76.

#### Medication adherence

The self-report Medication Adherence Report Scale (MARS-5) [13] consists of five items, each representing a different aspect of medication taking. Respondents indicate how often they engage in the five non-adherent behaviours, on a 1–5 frequency scale (always, often, sometimes, rarely, never). Higher scores indicate greater adherence to cardiac medication. A total score for all five items was calculated (range from 5 to 25). Scores were separated into unintentional non-adherence (item 1) and intentional non-adherence (items 2–5). The MARS-5 has demonstrated good reliability and validity [13], and it has been used widely in research as a measure of medication adherence behaviour [14–18]. In the study, the Cronbach’s alpha was 0.67 for the MARS-5 (total score).

#### Quality of life

Patients’ QoL was assessed using the Medical Outcomes Short-Form 12 (version 2) Health Survey (SF-12v2) [19], which is composed of 12 items. These are aggregated into two summary components; the physical component score (PCS) and mental component score (MCS). Rated items reflect what the individual is able to do functionally, how they felt and how they evaluated their health status. The SF-12v2 scores were calculated following the norm-based scoring algorithm, using weights derived from confirmatory factor analysis [20]. The measure has demonstrated good validity and reliability [19, 21]. In this study, the Cronbach’s alpha was 0.77 for the PCS and 0.81 for the MCS.

### Socio-demographic and clinical characteristics

Data were gathered on socio-demographics and the clinical characteristics of the participants. Occupation was identified in accordance with the Office of National Statistics categories (ONS 1998). The Carstairs index of social deprivation [22], provided deprivation categories based on postcode region of social deprivation in Scotland. Scores range from 1 to 7 and higher categories indicate greater deprivation (i.e. lower socio-economic status). Additionally, diagnosis, revascularisation, left ventricular ejection fraction, cardiac history, co-morbidity (i.e. hypertension, diabetes), other cardiovascular disease risk factors and current medications were identified from the patient’s clinical records.

### Statistical analysis

Changes in illness perceptions, beliefs about CR, medication adherence and QoL from baseline to 6 months were compared using the Wilcoxon Signed Ranks test. We investigated the strength of the linear relationships between the outcome variables i.e. medication adherence (MARS-5) and QoL (PCS, MCS) and independent variables i.e. illness perceptions (total score) and beliefs about CR (i.e. necessity, concerns about exercise, barriers and suitability), using Pearson’s Product Moment correlation. In order to assess whether differences in the self-reported outcomes i.e. medication adherence, physical and mental health at 6 months could be explained by differences in illness perceptions, beliefs about CR in patients starting a CR programme (baseline), we employed multiple logistic regression models on the outcomes, dichotomised as follows: MARS-5 was encoded at 0 for the group with the lower scores (under 25, sub-optimal adherence) and 1 for the group with a higher score (25, optimal adherence). Physical health (PCS) and mental health (MCS) were encoded 0 for the group with the lowest score (below 50) (i.e. poorer physical or mental health); and 1 for the group with higher scores (50 or above) (i.e. better physical or mental health), based on UK population means [23]. We fitted the model within the Bayesian framework [24, 25].

## Results

### Recruitment

Out of the patients approached upon entry to the CR programme, 56 patients consented to participate and completed the questionnaires at that time (baseline). At 6 months 40 (71%) of these patients completed the questionnaires and the data analysis was based on them.

### Characteristics of the participants

The socio-demographic and clinical characteristics of participants are presented in Table 1. Most patients were men (70%), mean age of 62.30 years (SD = 7.84); 22.5% had a diagnosis of STEMI, 52.5% with non-ST elevation myocardial infarction (NSTEMI) and the remainder either had unstable angina or stable angina. Most patients had a percutaneous coronary intervention (Table 1). Thirty patients (75%) completed the Bristol on-line survey and 25% completed paper copies of the questionnaire as their preferred method.

**Table 1:** Patients’ characteristics (*n* = 40)

Characteristics	Patients
Age, years (mean, range)	62.45 (45–78)
Male gender	28 (70%)
Marital status	
Married	37 (92.5)
Co-habilitating	3 (7.5%)
Employment	
Employed	20 (50%)
Unemployed or retired	20 (50%)
Education, years (median, range)	14.0 (7–30)
Social deprivation (SIMD)	
SIMD 1–2	10 (25%)
SIMD 3–5, out of area	30 (75%)
Diagnosis	
STEMI	9 (22.5%)
NSTEMI	21 (52.5%)
Unstable angina	5 (12.5%)
Stable angina	5 (12.5%)
Revascularisation	
Thrombolysis	2 (5%)
PCI	32 (80%)
CABG	1 (2.5%)
Left ventricular ejection fraction	
> 50%	21 (52.5%)
30–49% (mild - moderate impairment)	17 (42.5%)
< 29% (severe impairment), or missing	2 (5%)
Cardiac history	
PCI	5 (12.5%)
CABG	3 (7.5%)
Myocardial infarction	4 (10%)
Co-morbid conditions	
Hypertension	23 (57.5%)
Diabetes mellitus	2 (5%)
Other CVD risk factors	
Smoking	16 (40%)
Missing data	10 (25%)
Hypercholesterolaemia	21 (52.5%)
Missing data	2 (5%)
Medications	
ACE/ ARB	22/ 3 (62.5%)
Beta blocker	29 (72.5%)
Diuretics	2 (5%)
Antidepressants	6 (15%)

*SIMD* Scottish Index of Multiple Deprivation, *STEMI* ST elevation myocardial infarction, *NSTEMI* non-ST elevation myocardial infarction, *ACE* angiotensin converting enzyme inhibitor, *ARB* angiotensin receptor blocker

### Changes in illness perceptions, beliefs about CR, medication adherence and quality of life

Table 2 shows the changes in illness perceptions, beliefs about CR, medication adherence and QoL from baseline to 6 months later. Changes in illness perceptions (B-IPQ) (total scores) were statistically non-significant, but despite this the results indicate some negative illness perceptions with respect to the illness being treatable, higher levels of concern and general effect of illness on life which prevail over time. Perceived consequences (individual item B-IPQ) were statistically significantly reduced from baseline to 6 months, indicating the patients perceived fewer consequences of their disease (z = − 2.827, *p* = 0.005) (Table 2). Also, treatment control (z = − 2.132, *p* = 0.033) and illness concern (z = − 2.347, *p* = 0.019) were statistically significantly reduced at 6 months, which suggests that patients felt a greater sense that treatment could help their illness more and there were less concerns about their illness.. There was a trend for increased practical barriers (BCR-Q) at 6 months (z = − 1.905, *p* = 0.057), but the rest of the change scores for beliefs about CR were not statistically significantly different from baseline to 6 months (Table 2). The scores for necessity were high suggesting that patients were more likely to perceive CR as necessary and be clear as to how it would be of benefit. The scores for concerns about exercise were low suggesting that the patients were less concerned about participating in the exercise component of CR i.e. it may be harmful in some way. Similarly, the scores for practical barriers and perceived suitability were low indicating that the patients were less likely to perceive greater practical barriers to attending the CR programme and less likely to believe that CR is most suitable for a younger, more active person.

**Table 2:** Changes in patients’ illness perceptions, beliefs about cardiac rehabilitation, medication adherence and quality of life

Variable	Follow-up Median (range)	Baseline Median (range)	Change scores	% Change scores
Illness perceptions				
Consequences	2.00 (0–10)	4.00 (0–10)	− 2.00*	50.0
Timeline	10.00 (0–10)	9.00 (0–10)	1.00	11.1
Personal control	7.00 (0–10)	7.00 (0–10)	0.00	0.0
Treatment control	8.50 (2–10)	9.00 (4–10)	− 0.50*	5.5
Identity	2.00 (0–9)	2.50 (0–9)	− 0.50	20.0
Illness concern	4.50 (0–10)	6.50 (0–10)	− 2.00*	30.7
Coherence	8.50 (5–10)	9.00 (4–10)	− 0.50	5.5
Emotional upset	3.00 (0–9)	3.00 (0–10)	0.00	0.0
Total score	26.50 (4–56)	32.00 (10–55)	−5.50	17.2
Beliefs about CR				
Necessity	18.50 (10–21)	18.00 (13–21)	0.50	2.7
Concerns-exercise	5.50 (3–14)	6.00 (3–15)	−0.50	8.3
Practical barriers	5.00 (3–11)	4.00 (3–12)	1.00	25.0
Suitability	4.00 (2–8)	3.50 (2–10)	0.50	14.2
MARS-5 (total score)	25.0 (23–25)	25.0 (23–25)	0.00	0.0
Quality of life				
PCS	52.80 (23.94–62.72)	48.85 (19.08–60.26)	3.95	8.1
MCS	50.61 (24.05–62.25)	47.29 (29.98–61.88)	3.32	7.0

*CR* cardiac rehabilitation, *MARS-5* medication adherence report scale, *PCS* physical component score, *MCS* mental component score; *p = < 0.005

The medication adherence MARS-5 (total scores) were high at baseline and 6 months, indicating high levels of medication adherence (Table 2), which did not change statistically significantly over time. The unintentional non-adherence score was 4.70 mean (SD 0.56) at baseline and 4.70 mean (SD 0.51) at 6 months; and the intentional non-adherence was 20.0 mean (SD 0.0) at baseline and 6 months. There were no statistically significant changes in unintentional non-adherence (item 1) over time (25% vs 27.5%, *p* = 1.00); and also for intentional non-adherence (items 2 to 5) (0% vs 0%, *p* = 1.00). Table 2 shows the PCS and MCS scores at baseline and 6 months which indicate sub-optimal levels of physical and mental health, both of which prevailed over time.

### Correlations between illness perceptions, beliefs about CR, medication adherence and QoL

Table 3 shows that patients’ concerns about exercise (BCR-Q) at the start of CR were significantly weakly negatively correlated with their physical health (PCS) at 6 months (*r* = − 0.358, *p* = 0.023). This indicates that greater concerns about exercise were related to poorer physical health at 6 months. Also, patients’ poorer physical health and mental health at baseline were significantly weakly positively correlated with their poorer physical health (PCS) at 6 months (*r* = 0.327, *p* = 0.039 and *r* = 0.356, *p* = 0.024, respectively (Table 3). More negative illness perceptions (B-IPQ) and concerns about exercise (BCR-Q) at baseline were significantly weakly negatively correlated with poorer mental health (MCS) at 6 months (*r* = − 0.343, p 0.030 and *r* = − 0.457, *p* = 0.003, respectively) (Table 3). In addition, patients’ poorer mental health at baseline was significantly moderately positively correlated with their poorer mental health at 6 months (*r* = 0.596, *p* <  0.001). Table 3 shows the additional inter-correlations between variables.

**Table 3:** Correlations among medication adherence and quality of life and illness perceptions/beliefs about cardiac rehabilitation

		1	2	3	4	5	6	7	8	9	10	11
**Patients (n = =40)**												
1	MARS-5 (TP2)	1										
2	PCS (TP2)	−.01	1									
3	MCS (TP2)	−.01	.15	1								
4	Illness perceptions (BIPQ)	.02	.20	−.34*	1							
5	Necessity (BCRQ)	−.22	−.04	−.28	.27	1						
6	Exercise concerns (BCRQ)	−.05	−.35*	−.45*	.15	.06	1					
7	Barriers (BCRQ)	−.10	−.26	−.28	.17	−.14	.53**	1				
8	Suitability (BCRQ)	.07	−.15	−.07	−.02	−.17	.65**	.68**	1			
9	MARS-5	.29	.09	.01	−.11	−.11	−.11	−.23*	−.02	1		
10	PCS	−.19	.32*	.08	.27	−.01	−.32*	.09	−.02	−.01	1	
11	MCS	.20	.35*	.59**	−.42**	.35*	−.43*	−.20	−.40	.11	−.11	1

*MARS-5* Medication Adherence Report Scale, *B-IPQ* Brief Illness Perceptions questionnaire, *BCR-Q* Beliefs about cardiac rehabilitation questionnaire, *PCS* physical component score, *MCS* mental component score, *TP2* time-point 2

** *p* < 0 .01; *p < 0.05

### Impact of illness perceptions, beliefs about CR on medication adherence & QoL at 6 months

**Fig. 1:**
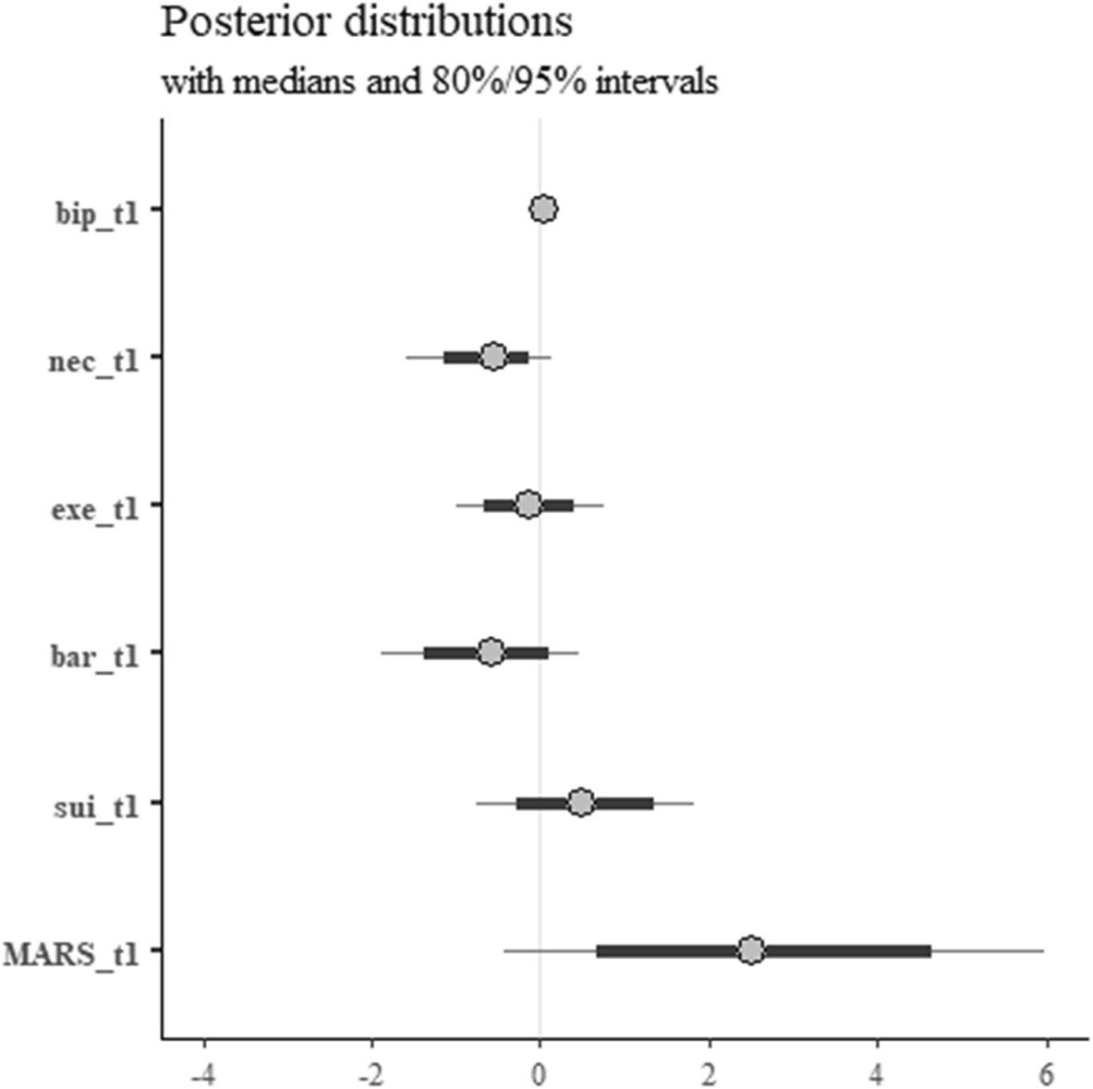
Posterior distributions for the model of medication adherence at 6 months. bip, illness perceptions; nec, perceived necessity of CR; exe, concerns about exercise; bar, practical barriers to CR; sui, perceived suitability of CR; MARS, Medication Adherence Report Scale

Inspection of Fig. 1 reveals that the model shows perceived suitability and MARS score at baseline (sui_t1 and MARS_t1 in the plot, respectively) to be positively related with the probability of scoring 1 on the 6-months dichotomised medication adherence (25 on the MARS scale), although with different magnitudes. The posterior distribution for the perceived suitability parameter presents most of its mass above 0, and is consistent with odds between 0.5–6 (point estimate 1.6); the baseline MARS parameter posterior distribution is almost entirely above zero, and is consistent with odds between 0.7–330 (point estimate 13.5).

Focussing on the point estimates, these can be interpreted as follows: keeping everything else at the mean level, an additional point on the perceived suitability scale increases the odds of being fully adherent of approximately 60%. On the other hand, being fully adherent at baseline increases the odds of staying so at 6 months by 13.5 times (as opposed to non-adherence at baseline). Perceived necessity, concerns for exercise, and practical barriers (nec_t1, exe_t1, and bar_t1 in the plot, respectively) are all associated to posterior distributions whose mass mostly lie below zero, although to different extents, meaning they can be interpreted as being overall negatively associated with the outcome. Specifically, necessity is consistent with odds between 0.2–1.1 (point estimate 0.5), concerns about exercise with odds between 0.4–1.2 (point estimate 0.9), and barriers with odds between 0.2–1.6 (point estimate 0.5). In terms of point estimates, these amount to reductions in probability of full adherence, all else being kept at the mean level, of 50, 10, and 50%, respectively for a one-point increase on those scales. Baseline illness perceptions (total score) seem to have a negligible impact on the outcome (odds between 0.9–1.2, point estimate 1.0).

**Fig. 2:**
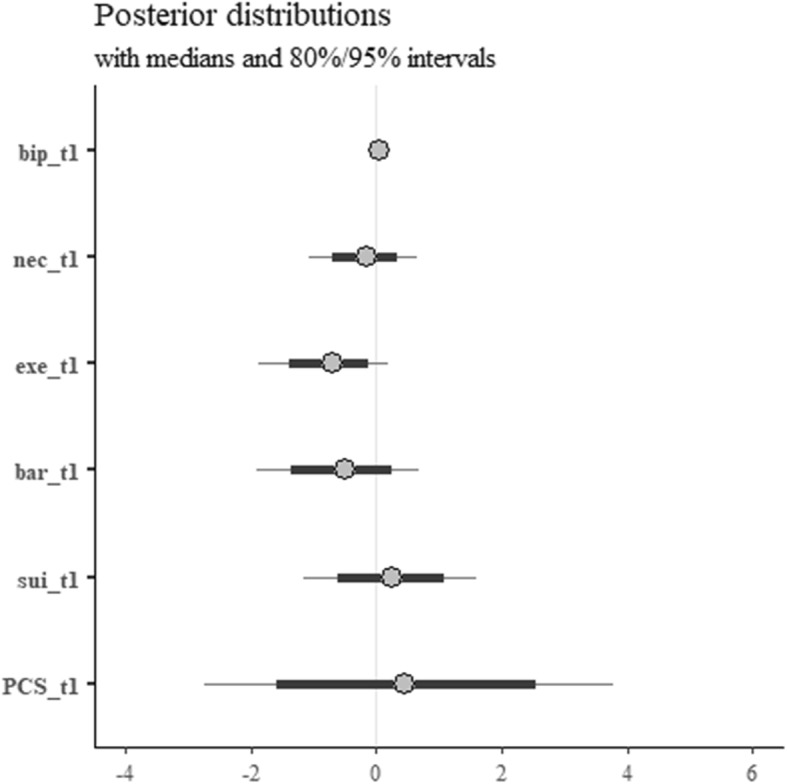
Posterior distributions for the model of physical health at 6 months. bip, illness perceptions; nec, perceived necessity of CR; exe, concerns about exercise; bar, practical barriers to CR; sui, perceived suitability of CR; MARS, Medication Adherence Report Scale

Figure 2 shows the posterior distributions for the model of physical health (PCS) at 6 months.

Physical health at 6 months was found to be negatively related to concerns for exercise and practical barriers at baseline (exe_t1 and bar_t1 in the plot, respectively). The posterior distributions for the concerns for exercise parameter lies almost entirely below 0, and is consistent with odds between 0.2–1.2 (point estimate 0.5); most of the distribution for the practical barriers parameter lies below zero, and is consistent with odds between 0.1–2.0 (point estimate 0.6). For what concerns the point estimates, these can be interpreted as follows: keeping everything else at the mean level, an additional point on the concerns for exercise scale decreases the odds of a PCS score higher than 50 of approximately 50%, while the same change on the practical barriers scale leads to a decrease in odds of approximately 60%. The posterior distributions of the parameters for perceived necessity, suitability, and physical health at baseline are all more or less centred around zero, thus making it difficult to assess the existence of an effect on physical health at 6 months. Baseline illness perceptions (total score) seem to have a negligible impact on the outcome (odds between 0.9–1.2, point estimate 1.0).

**Fig. 3:**
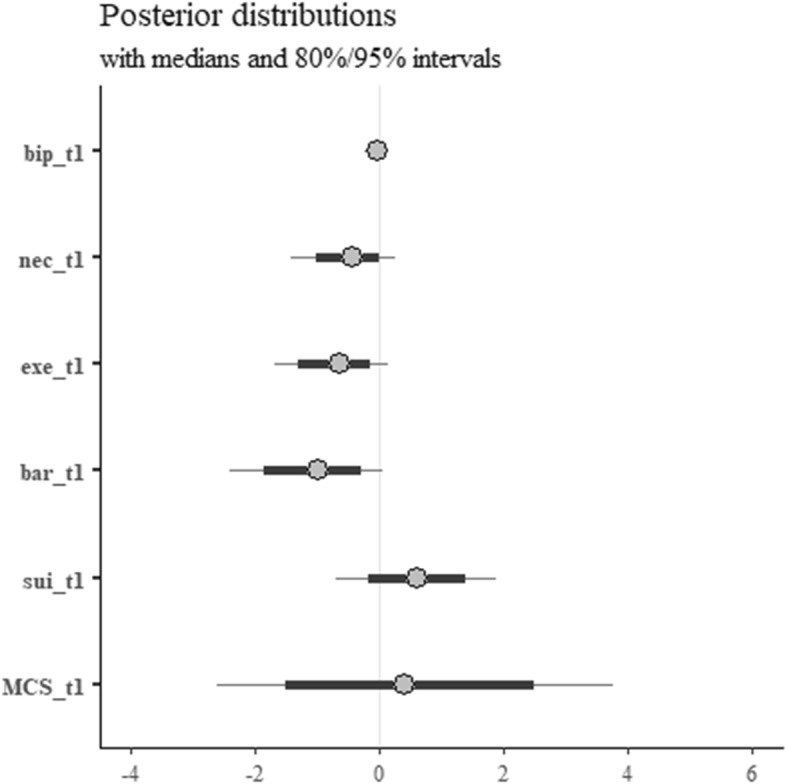
Posterior distributions for the model of mental health at 6 months. bip, illness perceptions; nec, perceived necessity of CR; exe, concerns about exercise; bar, practical barriers to CR; sui, perceived suitability of CR; MARS, Medication Adherence Report Scale

Figure 3 shows the posterior distributions for the model of mental health (MCS) at 6 months. Mental health at 6 months is found to be negatively related to perceived necessity, concerns for exercise and practical barriers at baseline (nec_t1, exe_t1 and bar_t1, respectively). The posterior distributions for all these parameters lie almost entirely below 0. An increase in perceived necessity at baseline is consistent with odds between 0.2–1.2 (point estimate 0.6); similarly, we observed that increases in concerns for exercise were consistent with odds between 0.2–1.1 (point estimate 0.5), and increases in practical barriers with odds between 0.1–1.1 (point estimate 0.4). In terms of point estimates, these can be interpreted as follows: keeping everything else at the mean level, an additional point on the scales related to perceived necessity, concerns for exercise, and practical barriers decrease the odds of an MCS score higher than 50 by approximately 60, 50, and 40% respectively, while the same change on the practical barriers scale leads to a decrease in odds of approximately 60%. Change on the practical barriers scale leads to a decrease in odds of approximately 60%. Perceived suitability is slightly positively related to the outcome, with a posterior distribution showing values consistent with odds between 0.5–6.7 (point estimate 1.8), interpretable as an 80% increase in the odds of an MCS score larger than 50 for a point increase in perceived suitability. While baseline illness perception (total score) does not exhibit any evident impact on MCS at 6 months, the posterior distribution for baseline MCS scores is consistent with too wide a range of odds (0.1–40.4, point estimate 1.5) to help draw reliable conclusions.

## Discussion

This study examined whether differences in self-reported outcomes i.e. medication adherence, physical and mental health at 6 months could be explained by differences in illness perceptions and beliefs about CR upon entry to a CR programme. The results suggest that small changes (i.e. improvements) in perceived suitability i.e. beliefs about CR increase the probability of being fully adherent by approximately 60%. Also, being fully adherent to medication upon entry to a CR programme increases the odds of staying so at 6 months by 13.5 times. Perceived necessity, concerns regarding exercise and practical barriers to CR were negatively associated with reductions in the probability of full medication adherence. Previous research has examined beliefs about CR although mostly in relation to CR attendance [11, 26, 27]. Our results suggest that patients’ beliefs about the suitability of CR and medication adherence at the start of CR are especially important to medication adherence at 6 months. Medication adherence should be addressed early as part of the CR process as well as any mistaken beliefs about CR. In our study, patients’ baseline illness perceptions showed no real impact on medication adherence at 6 months, a finding which is largely consistent with Byrne et al. [28]. Although illness perceptions and CR attendance and/or secondary prevention have been examined [9, 27, 29], there remains a paucity of evidence on the relation between illness perceptions and medication adherence (outcome) in CAD patient populations.

Our analysis suggests that physical health (outcome) at 6 months is negatively related to baseline concerns about exercise and practical barriers [12, 27]. These results are broadly consistent with Cooper et al. [11], who assessed beliefs about CR as a basis for predicting CR attendance after acute MI. As yet, there is a scarcity of research examining the associations between beliefs about CR and physical health (outcome), Prior research has shown that poorer health outcomes for ACS patients may be attributed to non-attendance at, or non-completion of CR and that high scores for perceived necessity following discharge predict attendance at CR [26]. It was unclear in our analysis whether poorer physical health at 6 months was influenced by perceptions of necessity and suitability, and physical health at baseline. In addition, we found no real impact of baseline illness perceptions on physical health at 6 months, which contrasts with previous research [7, 8, 30–34].

Mental health (outcome) at 6 months was negatively associated with perceived necessity, concerns for exercise and practical barriers at baseline. No studies were identified for direct comparison with our results for mental health, although concerns about the harmful effects of CR are said to reflect an emotional reaction [11]. Prior research [11, 35], has identified that patients who hold negative beliefs about CR were less likely to attend CR. Our correlation analysis revealed that greater baseline concerns about exercise and negative illness perceptions were significantly related to poorer mental health (outcome) at 6 months. Prior research has established that patients’ treatment beliefs e.g. beliefs about CR are not necessarily isolated from their illness beliefs, although mental health was not examined [36].

A further aim of the study was to compare changes in medication adherence, perceived physical and mental health, illness perceptions and beliefs about CR upon entry to a CR programme and 6 months later. Despite our study showing no statistically significant changes in medication adherence (MARS) upon entry to the CR programme to 6 months later, it revealed some important information. The baseline and 6 months scores were high suggesting greater medication adherence; a finding which is consistent with prior studies with cardiac patients [28], and stroke survivors [16, 17]. In this study, 75% of patients reported optimal adherence and 25% of patients reported sub-optimal adherence at baseline; and 72.5% of patients reported optimal adherence and 27.5% of patients reported sub-optimal adherence at 6 months. These figures reveal greater medication adherence compared to previous research [14]. In our study, 25% of patients reported unintentional non-adherence at baseline and 27.5% of patients at 6 months, compared to 15% at 2 weeks and 52% at 6 months in Molloy et al. [14]. In our study, the patients stating intentional non-adherence was zero at baseline and 6 months, which is low compared to prior research [14]. Still our results suggest some unintentional non-adherence to cardiac medications which should be avoided. Medication adherence is crucial for successful CR [18], and the prevention of recurrent MI and premature mortality [1].

There were no statistically significant changes in physical health (PCS) and mental health (MCS) over time. The scores remained below the population average of 50 [37], which is consistent with other cardiac studies [38–40]. Our results imply no real impact of CR on QoL, a finding which is consistent with the RAMIT trial [41]. The evidence, however, is conflicting with previous studies, reviews and meta-analyses claiming the benefit for QoL with CR [42, 43]. McKee [44] argues that improvements in QoL mainly occur during the CR programme phase. The patients in our study attended CR classes 1 day per week for 10 weeks. The study was not designed as a trial of the ‘efficacy’ of CR but rather it sought to help clarify the likelihood of increased medication adherence and better QoL at 6 months having considered patients’ illness perceptions and beliefs about CR at baseline.

Similar to Jones et al. [26], we found, for example, that perceived necessity of CR did not change significantly over time, although the baseline scores were high compared to other research [11], suggesting our patients were more likely to perceive CR as necessary and beneficial. Consistent with prior research [33], our patients reported fewer consequences of their illness over time, a greater sense of control over their cardiac disease and less concerns about illness. Compared to attenders at CR [27], our total B-IPQ scores were high reflecting a more threatening (negative) view of illness. These dissimilar findings may be due to the different timing of assessment. The scores for concerns about exercise and perceived suitability at baseline were low compared to Cooper et al. [11], indicating our patients were less concerned about the exercise component of CR and less likely to believe that it was more suitable for a younger more active person. Despite these findings, there were still some erroneous beliefs about CR suggesting some room for improvement. Individuals may be less likely to continue participating in CR if incorrect beliefs about CR are not targeted [11, 33].

### Strengths and limitations

Patients were recruited from a standard CR service, but this was a relatively small convenience sample of CAD patients which may limit generalisability to the wider UK population. Strengths of the study lie in its longitudinal design and in the selection of antecedent variables (i.e. illness perceptions and beliefs about CR) that to our knowledge have not been used before in combination to assess associations with medication adherence and QoL outcomes at 6 months. Individual items on the B-IPQ were not analysed separately in the logistic regression and this may have obscured some significant findings. Given the overall high medication adherence scores there may have been some reporting bias. However, the wording of the MARS has been shown to reduce a social desirability effect [17], and the measure has been shown to correspond to more objective measures of adherence such as the Medication Event Monitoring System (MEMS) [45], and it is considered to be more practical in the rehabilitation setting [46]. Overall, beliefs about CR featured highly in this study with respect to medication adherence and QoL. Considering the uniqueness of these results more research is needed using a larger sample size to confirm or refute our findings.

## Conclusion

Our results suggest that patients’ beliefs at the start of a CR programme are especially important to medication adherence at 6 months. In addition, being fully adherent to medication upon entry to a CR programme increases the odds of staying so at 6 months. Physical and mental health at 6 months was negatively associated with baseline beliefs about CR. Negative beliefs about CR should be identified early as part of CR to counteract any negative effects on QoL. Interventions to improve medication adherence and QoL outcomes should focus on improving patients’ negative beliefs about CR and increasing understanding of the role of medication adherence in preventing a future cardiac event.

## Data Availability

The datasets used and/or analysed during the current study are available from the corresponding author on reasonable request.

## Abbreviations

ACS: Acute coronary syndrome
BCR-Q: Beliefs about cardiac rehabilitation questionnaire
B-IPQ: Brief illness perception questionnaire
CAD: Coronary artery disease
CR: Cardiac rehabilitation
MARS: Medication adherence report scale
MCS: Mental component score
MI: Myocardial infarction
NSTEMI: Non-ST elevation myocardial infarction
PCS: Physical component score
QoL: Quality of life
SF-12v2: Medical outcomes short-form 12 (version 2) health survey
STEMI: ST elevation myocardial infarction
